# Development and validation of a highly sensitive HPLC method for quantifying cardiovascular drugs in human plasma using dual detection

**DOI:** 10.1038/s41598-025-94907-0

**Published:** 2025-04-10

**Authors:** Khalid A. M. Attia, Ahmed H. Abdel-Monem, Ahmed M. Abdel-Raoof, Amr S. Eissa

**Affiliations:** 1 Pharmaceutical Analytical Chemistry Department, Faculty of Pharmacy, Al-Azhar University, Nasr City, Cairo 11751 Egypt; 2 Pharmaceutical Chemistry Department, Faculty of Pharmacy, Egyptian Russian University, Badr City, Cairo 11829 Egypt

**Keywords:** Cardiovascular drugs, Human plasma, HPLC, Fluorescence, Liquid liquid extraction, Analytical chemistry, Bioanalytical chemistry

## Abstract

Cardiovascular diseases are the major cause of global mortality, and often require the concomitant use of a number of drugs to prevent and reduce these deaths. The challenge is to find effective and accurate methods for analyzing these drugs in plasma. This research introduces an innovative, sustainable HPLC-FLD method for the concurrent determination of bisoprolol (BIS), amlodipine besylate (AML), telmisartan (TEL), and atorvastatin (ATV) within human plasma. Chromatographic separation was achieved using an isocratic elution mode on a Thermo Hypersil BDS C18 column (150 × 4.6 mm, 5.0 μm), while the mobile phase comprised of ethanol and 0.03 M potassium phosphate buffer (pH 5.2) in a 40:60 ratio, with a flow rate of 0.6 mL/min. The eluate was analyzed using UV detection within the wavelength range of 210–260 nm to confirm effective separation of the four cardiovascular drugs. For enhanced specificity, a fluorescence detector was set to 227ex/298em for BIS, 294ex/365em for TEL, 274ex/378em for ATV, and 361ex/442em for amlodipine. The method was validated following the International Council for Harmonisation (ICH) guidelines. Linearity was established within the ranges of 5–100 ng/mL for BIS and AML, 0.1–5 ng/mL for TEL, and 10–200 ng/mL for ATV, ensuring accuracy and precision. The significant of the current work represented in introduction of a highly sensitive, and selective analytical method, utilizing an economical sample preparation strategy, for the simultaneous determination of four different cardiovascular drugs (bisoprolol, amlodipine, telmisartan, and atorvastatin) in spiked human plasma. The extraction of sample was carried by liquid-liquid extraction (LLE) and analyzed by LC-fluorescence detector. The chromatographic run was short (less than10 min) which is a greet economical value.

## Introduction

Hypertension is the most complicated disease as it increases the risk of many different diseases such as cognitive impairment, dementia, left ventricular hypertrophy, myocardial ischemia, stroke, congestive cardiac failure, chronic kidney failure, encephalopathy, heart failure, and coronary artery disease^1–3^. All of these complications lead to an increase in the mortality rate in hypertensive patients rather than normal^4^. Moreover, Coronaviruses (CoVs) are a class of RNA viruses characterized by being of different specie with a continuous mutation which produce their negative impact on respiratory, GIT, hepatic, and nervous system^5^. Evidence-based clinical trials reveal that hypertension individuals are at a higher risk of coronavirus infection, with longer-lasting symptoms that increase mortality^6^. As a result, medication monitoring for hypertensive patients is mandatory in normal and especially during Covid-19 propagation.

Hypertension is clinically recognized when systolic blood pressure exceeds 140 mmHg or diastolic pressure surpasses 90 mmHg^7^. Its management typically involves five key drug categories: diuretics, calcium channel blockers, beta-blockers, angiotensin-converting enzyme inhibitors, and angiotensin receptor blockers^8^. One of the most critical consequences of hypertension is its potential to induce endothelial dysfunction, thereby accelerating the atherosclerotic process and promoting the formation of unstable atherosclerotic plaques^9^.

**Fig. 1:**
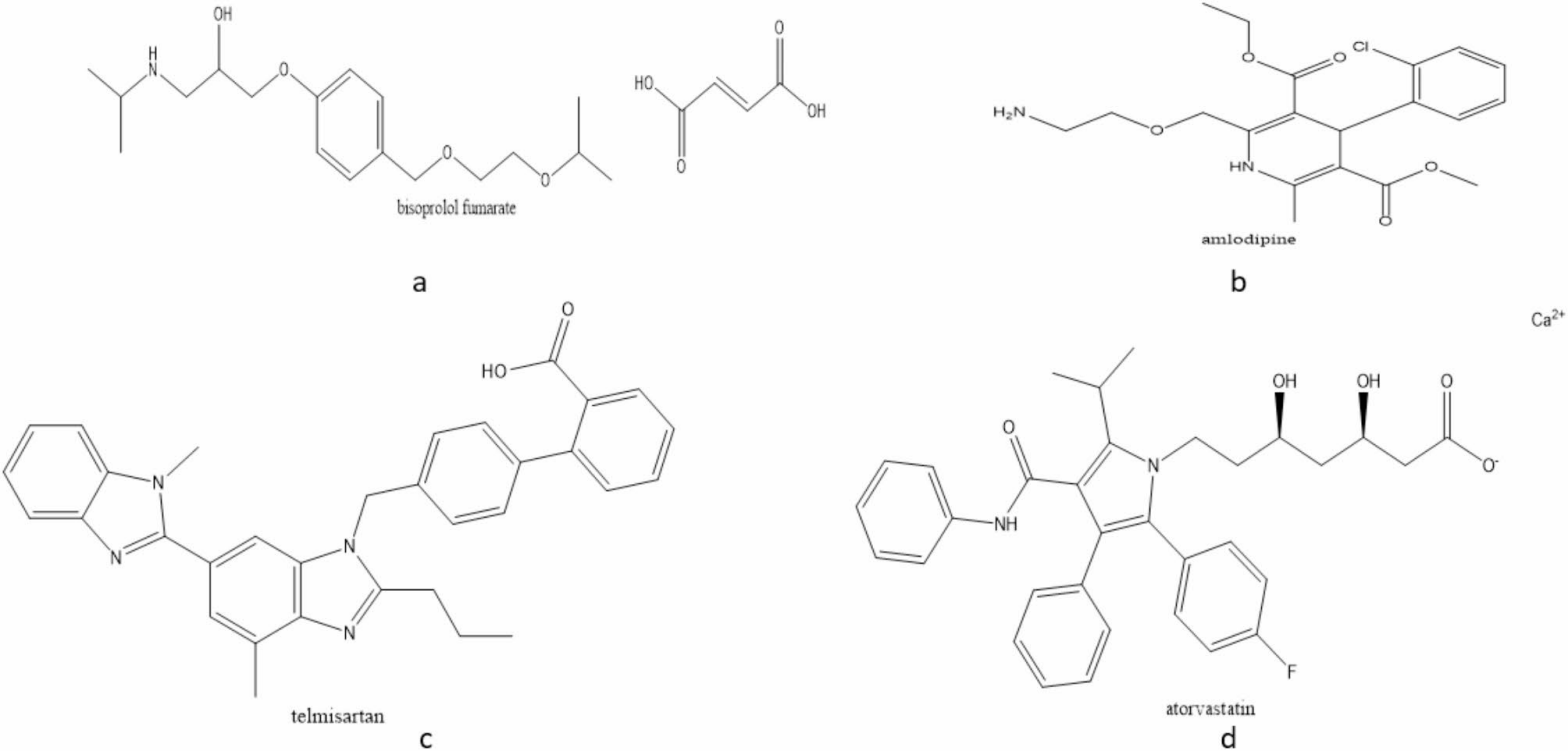
(**a**) Chemical structure of bisoprolol fumerate, (**b**) chemical structure of amlodipine, (**c**) chemical structure of telmisartan, (**d**) chemical structure of atorvastatin.

Bisoprolol fumarate, a selective beta-1 adrenergic receptor blocker, is commonly prescribed for managing moderate to severe hypertension in humans, it is soluble in ethanol, methanol, and water^10^, its chemical structure is shown in (Fig. 1a). Amlodipine, a dihydropyridine derivative, works by altering calcium ion movement, interacting with the sarcoplasmic reticulum, and inhibiting calcium influx through voltage-dependent channels. This dual action targets both peripheral vascular and coronary smooth muscle cells^11^, and its chemical structure is shown in (Fig. 1b). Telmisartan acts by preventing angiotensin II from binding to its receptors, blocking vasoconstriction, reducing aldosterone production, and preventing renal reabsorption, thus lowering blood pressure^12^, its chemical structure is shown in (Fig. 1c). Finally, atorvastatin calcium trihydrate, a cornerstone in atherosclerosis treatment, reduces cholesterol production by inhibiting HMG-CoA reductase in the liver. This promotes the increase of LDL receptors, reducing LDL cholesterol levels^13^, its chemical structure is illustrated in (Fig. 1d).

According to the 2020 International Society of Hypertension Global Hypertension Practice Guidelines: Hypertension management involves one or more cardiovascular drugs, selected based on hypertension severity and associated risk factors. These antihypertensive agents are among the most frequently prescribed, either as standalone therapies or in fixed-dose combinations containing at least two of them^14^, as Concor 5 mg^®^, Concor Amlo 5/5 ^®^, Norvasc 5 mg^®^, twynsta 80/10^®^, caduet 10/20^®^, and telista 40 mg^®^ which are used for management of moderate to severe hypertension. The literature review of these drugs as mixture revealed different spectrophotometric methods^15–28^, spectrofluorimetric^29–34^, and chromatographic methods^35–41^.

The goal of this study is to establish a simple, accurate, and rapid method for monitoring the plasma concentrations of four cardiovascular drugs: angiotensin receptor blockers (telmisartan), beta-blockers (bisoprolol), calcium channel blockers (amlodipine), and antihyperlipidemic agents (atorvastatin).

## Experimental

### Chemical and reagent

BIS was generously provided by Pharco Pharmaceuticals, Cairo, Egypt, while ATV was kindly supplied by E.I.P.C.O., Cairo, Egypt. TEL and AMLwere given by Parkville Pharmaceuticals, Cairo, Egypt. The purities of these standards were certified to be 99.24, 99.13, 99.69, and 99.78% for BIS, ATV, TEL, and AML, respectively.

Double-distilled water was prepared using Aquatron automatic water stills (Stuart, UK). Ethanol, diethyl ether, dichloromethane, and potassium dihydrogen phosphate were purchased from Sigma-Aldrich, Germany. Human plasma samples were provided from VACSERA, Cairo, Egypt.

### Instrumentation and chromatographic conditions

Quantification and analysis of the cardiovascular drugs were carried out using a Waters Alliance 2695 HPLC system (Waters, USA) equipped with an auto-sampler injector, solvent cabinet, and a quaternary pump. Detection was conducted using a Waters 2996 photodiode array detector, equipped with a standard flow cell (10 mm path length, 1000 psi maximum pressure), and T 50 Waters 2475 Multi-Wavelength Fluorescence Detector (Waters, USA) with standard quartz flow cell (10 mm path length, 1000 psi maximum pressure), excitation wavelength range 200–800 nm, emission wavelength range 210–900 nm.The chromatographic separation was carried out on a Thermo Hypersil BDS C18 column (150 mm × 4.6 mm, 5 μm).

Evaporation of plasma samples was facilitated by a rotary evaporator (Scilogex, USA). The pH of solutions was carefully regulated using a Jetway 3505 pH meter (England). Plasma samples were centrifuged using a Centurion Scientific Ltd^®^ centrifuge (Hunan, China) with a maximum speed of 6000 rpm.

An isocratic elution approach was used to achieve optimal separation, employing a mobile phase of ethanol and potassium dihydrogen phosphate buffer (0.03 M KH_2_PO_4_, pH 5.2) in a 40:60 ratio. The flow rate was maintained at 0.6 mL/min, with a consistent injection volume of 20 µL. The column oven temperature was carefully regulated between 25 and 35 °C. UV scanning was utilized to monitor the eluates within the 210–260 nm wavelength range and the detection was carried on 240 nm, ensuring precise separation and accurate identification of the four cardiovascular drugs.

Fluorescence detection settings were optimized for enhanced sensitivity, with excitation/emission wavelengths adjusted to 227/298 nm for BIS, 294/365 nm for TEL, 274/378 nm for ATV, and 361/442 nm for AML. Instrument parameters were fine-tuned to support simultaneous detection of all analytes. To prevent cross-contamination, the autosampler was thoroughly rinsed with the mobile phase for 10 min after each injection. Data acquisition and analysis were carried out using Waters Alliance 2695 software, ensuring accuracy and reliability in the results.

### Standard solutions

Stock solutions of each cardiovascular drug (100 µg/mL) were prepared by dissolving 10 mg of each drug in 50 mL of ethanol, and the volume was then adjusted to 100 mL with the same solvent. Working solutions (10 µg/mL) were prepared by further diluting the stock solutions to the required concentration. The prepared standards were kept at 2–8 C^0^ and has shown a stability for three weeks.

### Sample extraction

The drugs were extracted from plasma using a two-step liquid-liquid extraction (LLE) technique. Initially, 600 µL of absolute ethanol was added to 200 µL of plasma and 50 µL of the working standard solution. After vortexing, the mixture was centrifuged for 2 min to remove proteins. Then, 1.0 mL of diethyl ether (first extraction solvent) was added, followed by vortexing for 5 min and centrifugation at 3500 rpm for 5 min at 0 °C. The organic phase was carefully separated and transferred to a clean test tube.

In the second extraction step, 0.5 mL of dichloromethane was added as the second extraction solvent, followed by vortex mixing for 5 min and centrifugation at 3500 rpm for 5 min at 0 °C. The organic layer was carefully collected and combined with the layer obtained from the first extraction. The combined organic layers were then evaporated under a gentle stream of nitrogen at 40 °C. Once evaporation was complete, the residue was reconstituted in 500µL of ethanol, vortexed for 2 min, and 20 µL of the prepared solution was injected into the HPLC system for analysis^42^.

### Method validation

The validation of the proposed method was performed following the guidelines outlined by the International Council of Harmonization (ICH) for bioanalytical method validation^43^. Key aspects of the validation included assessing the calibration curve, linearity, lower limit of quantification (LLOQ), matrix effect (ME), extraction recover (ER), selectivity, accuracy, precision, stability, and system suitability.

## Results and discussion

Reverse-phase high-performance liquid chromatography (RP-HPLC) is widely utilized in the pharmaceutical industry for efficient separation and precise quantification of active pharmaceutical ingredients in dosage forms. Moreover, it has been incorporated in environmental, plasma, and veterinary use. This addition of use is due to its efficacy, accuracy, precision, reproducibility, and ability to quantify nano concentrations^44^. HPLC has different types of detectors such as UV spectrophotometer, fluorescence, refractive index, mass spectrometer, and electrochemical detector which afford the analysis of different types of samples in different fields^45^. The most used detector for drug quantification in plasma is the mass spectrometer detector, on the contrary, the mass spectrometer detector has the limitation of not distinguishing between hydrocarbons that have the same fragments, availability of the device in the market, and expensive instrumentation method in the Egyptian market. On the other hand, fluorescence detector has the privilege of selectivity and specificity to quantify low concentrations of the drug but it is limited to fluorescence compounds only^46^.

### Optimization of experimental conditions

For determination of these cardiovascular drugs using an isocratic elution and a reversed-phase Thermo Hypersil BDS C18^®^ column, various mobile phase compositions were tested, including mixtures of ethanol, sodium dodecyl sulfate, and phosphate buffer. However, issues such as peak tailing, splitting, and ineffective separation were encountered. The optimal mobile phase, consisting of phosphate buffer (pH 5.2) and ethanol, provided the best separation, yielding sharp peaks and high sensitivity within a short analysis time.

**Fig. 2:**
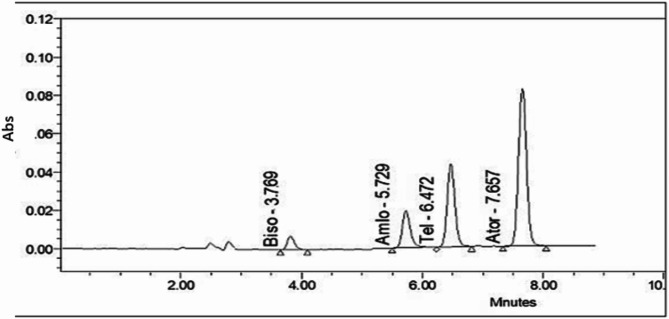
Chromatographic separation of bisoprolol, amlodipine, telmisartan, and atorvastatin (10 µg/ml) at λ 240 nm.

**Fig. 3:**
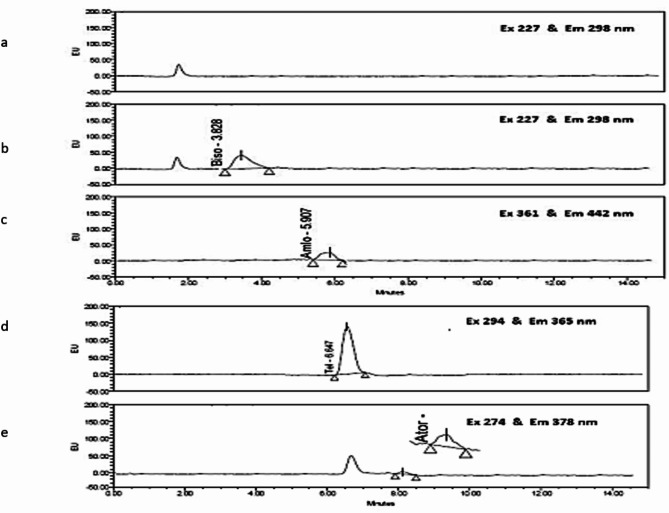
Chromatographic separation of selected drugs at different excitation and emission wavelengths (**a**) for blank plasma (**b**) bisoprolol 30 ng/mL (**c**) amlodipine 30 ng/mL, (**d**) telmisartan 5 ng/mL, and (**e**) atorvastatin 50ng/mL.

Various flow rates of 0.2, 0.6, 0.8, and 1 mL/min were evaluated, 0.6 mL/min was determined to be the most effective, providing optimal separation without peak distortion. Initial separation was verified using UV detection, as depicted in (Fig. 2). Subsequently, fluorescence detection was performed, with excitation/emission wavelengths set to 227/298 nm for BIS, 294/365 nm for AML, 274/378 nm for TEL, and 361/442 nm for ATV. After fine-tuning the chromatographic conditions, well-resolved and symmetrical peaks were obtained, as illustrated in (Fig. 3a–e).

### Drug extraction

Drugs extraction was the problematic step in the separation as the degree of binding of these drugs to the plasma protein reached more than 98% ^47–50^, so the normal extraction methods as addition of acetonitrile or ethanol as one step was not adequate for proper extraction and separation for the four drugs. Various extraction methods have been carried out; the two-step liquid-liquid extraction method has been shown to be the proper method for extracting these drugs^42^. The two-step liquid-liquid extraction method depends firstly on the addition of absolute ethanol for precipitation of the plasma protein, then addition of two different organic solvents in two separate steps to ensure complete extraction of the four drugs. Subsequently, complete evaporation of the organic solvent under low temperature with a rotary evaporator till dryness. To finish, the dried solution was reconstituted with 500 µL of ethanol.

### Method validation

The proposed method was validated in compliance with the guidelines of the International Council of Harmonisation (ICH) for bioanalytical validation^51^. The validation process utilized human plasma to ensure the method’s reliability for determining plasma drug levels in patients receiving these medications. Reported maximum plasma concentrations following administration were as follows: 9.057 ± 2.267 ng/mL for 10 mg of AML^52^, 507 ± 100 ng/mL for 80 mg of TEL^53^, 31 ng/mL for 5 mg of BIS^54^, and 3.61 µg/mL for 10 mg of ATV^48^.

Key validation parameters assessed included calibration curve linearity, lower limit of quantification, matrix effect, extraction recover, selectivity, accuracy, precision, stability, and system.

#### Linearity and range

Calibration curves were constructed for each drug by plotting peak areas against their respective concentrations (ng/mL) using the outlined experimental conditions. The analysis demonstrated linearity within the ranges of 5–100 ng/mL for BIS and AML, 0.1–5 ng/mL for TEL, and 10–200 ng/mL for ATV. Detailed regression equations, along with intercepts, slopes, and correlation coefficients, are provided in (Table 1). The high correlation coefficients and minimal intercept and slope values confirmed the excellent linearity of the calibration curves, ensuring accuracy and consistency within the specified ranges.

**Table 1:** Regression and validation data for determination of Bisoprolol, amlodipine, Telmisartan, and Atorvastatin by the proposed HPLC-FLD method.

Parameters	Bisoprolol	Amlodipine	Telmisartan	Atorvastatin
Linearity range (ng/mL)	5–100		0.1–5	10–200
Regression equation	y* = bx** +* a*			
Slope (b)	104061.01	16668.45	9026302.91	2990.89
Intercept (a)	−125370.42	156206.08	1599442.86	6912.77
LLOQ (ng/mL)	5	2	0.03	10
Coefficient of determination (r^2^)	0.9992	0.9997	0.9996	0.9992
Robustness				
Effect of ethanol (±1%)	94.59 ± 2.36	93.54 ± 3.44	94.07 ± 3.21	91.02 ± 3.36
Effect of temp ((±1 C)	90.12 ± 3.25	94.83 ± 3.07	95.26 ± 2.43	92.87 ± 2.36
Effect of pH (±0.1)	90.99 ± 1.89	93.71 ± 2.99	94.07 ± 1.21	96.02 ± 3.36

#### Lower limit of quantification (LLOQ)

The lower limit of quantification of the proposed method was evaluated by determining five replicates of lowest expected concentration than can be assessed accurately and precisely. Detailed results are provided in (Table 1).

#### Accuracy

The accuracy of the proposed method was determined by comparing the measured concentrations of the drugs with their known true values for three quality control samples (LQC, MQC, HQC). The results, presented as the average percentage recovery ± standard deviation, are summarized in (Table 2), confirming the method’s high level of accuracy and reliability.

**Table 2:** Accuracy and precision results for determination of Bisoprolol, amlodipine, Telmisartan, and Atorvastatin by the proposed HPLC-FLD detector method.

			Repeatability		Intermediate	
	QC level	QC concentration (ng/ml)	% recocery ± SD	%CV	% recocery ± SD	% CV
**Bisoprolol**	LLOQ	5	100.33±0.105	0.10374	98.866±0.090	0.0912
	LQC	10	98.33±0.611	0.621	93.66±0.152	0.163
	MQC	50	98.33±1.011	1.029	103.81±4.497	4.333
	HQC	90	101.11±2.001	1.978	97.77±4.35	4.458
**Amlodipine**	LLOQ	2	101.66±0.076	0.0751	106.666±0.208	0.195
	LQC	10	97.33±0.611	0.627	93.00±0.264	0.284
	MQC	50	100.66±2.081	2.067	94.00±3.001	3.191
	HQC	90	97.77±2.645	2.706	95.925±3.214	3.351
**Telmisartan**	LLOQ	0.05	90.01±0.001	0.001	96.66±0.004	0.0036
	LQC	0.1	94.33±0.004	0.0042	97.33±0.011	0.011
	MQC	0.25	90.66±0.031	0.0336	90.66±0.025	0.028
	HQC	0.4	100.83±0.047	0.047	85.83±0.040	0.047
**Atorvastatin**	LLOQ	10	106.66±1.527	1.432	96.66±2.081	2.153
	LQC	30	94.44±3.511	3.718	104.44±3.055	2.925
	MQC	100	92.66±2.516	2.715	101.66±3.511	3.454
	HQC	180	97.77±3.605	3.687	95.93±2.516	2.623

#### Precision

The precision of the proposed method was assessed at two levels: repeatability and intermediate precision. Repeatability was evaluated by analyzing the samples three times within a single day, while intermediate precision involved measuring the samples on three different days. The results were presented as coefficient of variation (% CV), as summarized in (Table 2), indicating the method’s consistent performance.

#### Matrix effect

To investigate the influence of endogenous plasma compounds on analyte response, Matrix effect was tested using two distinct sample sets. The first set contained samples without plasma, while the second included plasma samples after the extraction of cardiovascular drugs at three different quality control levels. The results were expressed as percentage of matrix effect, the findings are detailed in (Table 3).

**Table 3:** Extraction recovery and matrix effect data for determination of Bisoprolol, amlodipine, Telmisartan, and Atorvastatin by the proposed HPLC-FLD method.

	QC level	QC concentration (ng/ml)	% ER ± RSD	% ME
**Bisoprolol**	LQC	10	98.77 ± 0.435	99.52
	MQC	50	100.72 ± 0.056	92.31
	HQC	90	104.82 ± 3.511	96.04
**Amlodipine**	LQC	10	103.33 ± 0.702	94.35
	MQC	50	105.33 ± 2.081	101.42
	HQC	90	105.18 ± 3.958	93.67
**Telmisartan**	LQC	0.1	102.32 ± 0.098	91.19
	MQC	0.25	104.66 ± 0.763	95.67
	HQC	0.4	109.16 ± 0.015	96.85
**Atorvastatin**	LQC	30	102.03 ± 3.214	94.67
	MQC	100	104.33 ± 3.785	98.63
	HQC	180	107.77 ± 0.577	93.05

#### Efficacy rate

The efficacy of the extraction method in isolating analytes from the matrix was evaluated using two primary sample sets. The first set consisted of pre-extraction samples, while the second included plasma samples following the extraction of cardiovascular drugs at three different quality control levels. The results were expressed as percentage of efficacy rate ± standard deviation as presented in (Table 3).

#### Robustness

The robustness of the proposed method was tested by making small alteration to critical parameters, such as pH (± 0.1), ethanol concentration (± 1%), and temperature (± 1 °C), while keeping all other conditions unchanged. Each trial involved altering only one parameter at a time. The low %RSD values obtained indicated the method’s reliability and resilience under slight variations, as detailed in (Table 1).

#### System suitability

Moreover, system suitability parameters including (retention time, USP platelet count, capacity factor, and USP tailing factor) are shown in (Table 4).

**Table 4:** System suitability parameters for Bisoprolol, amlodipine, Telmisartan and Atorvastatin using the proposed HPLC-FLD method.

Parameter	Bisoprolol	Amlodipine	Telmisartan	Atorvastatin	Normal value
Retention time	3.828	5.914	6.64	8.079	
USP platelet count	2008	3465.706	5267.4	4736.16	>2000
Capacity factor (K’)	1.32	2.28	2.712	3.455	>1
USP tailing factor	1.06	1.102	1.27	1.21	0.9–2.0

### Plasma application

Adherence to prescribed medication significantly contributes to reducing mortality^14^, which is further supported by continuous drug monitoring in human plasma. The proposed method facilitates the accurate determination of drug concentrations in plasma, with Cmax values falling within the linear range for each drug. As detailed in (Table 5), the regression equation was used to calculate the concentrations of the drugs.

**Table 5:** Application of the proposed method for analysis of Bisoprolol, amlodipine, Telmisartan, and Atorvastatin in human plasma using the proposed HPLC-FLD method.

Laboratory mixture (ng/ml)				Recovery %			
Bisoprolol	Amlodipine	Telmisartan	Atorvastatin	Bisoprolol	Amlodipine	Telmisartan	Atorvastatin
10	10	0.15	15	98.2	95.89	100.21	98.09
30	30	2	30	99.5	97.85	101.4	95.23
50	50	3	100	97.8	99.89	99.67	97.78
80	80	4	150	100.5	98.56	99.21	96.16
90	90	0.45	189	98.02	99.79	100.32	99.88
Mean				98.80	98.40	100.16	97.43
S.D.				1.034	1.468	0.736	1.613

### Green assessment

Green analytical chemistry (GAC) plays a crucial role in sustainable development by reducing the environmental and health risks associated with analytical processes. By minimizing hazardous substances, waste generation, and energy consumption, it ensures analytical precision while promoting safety and sustainability^55^.

**Fig. 4:**
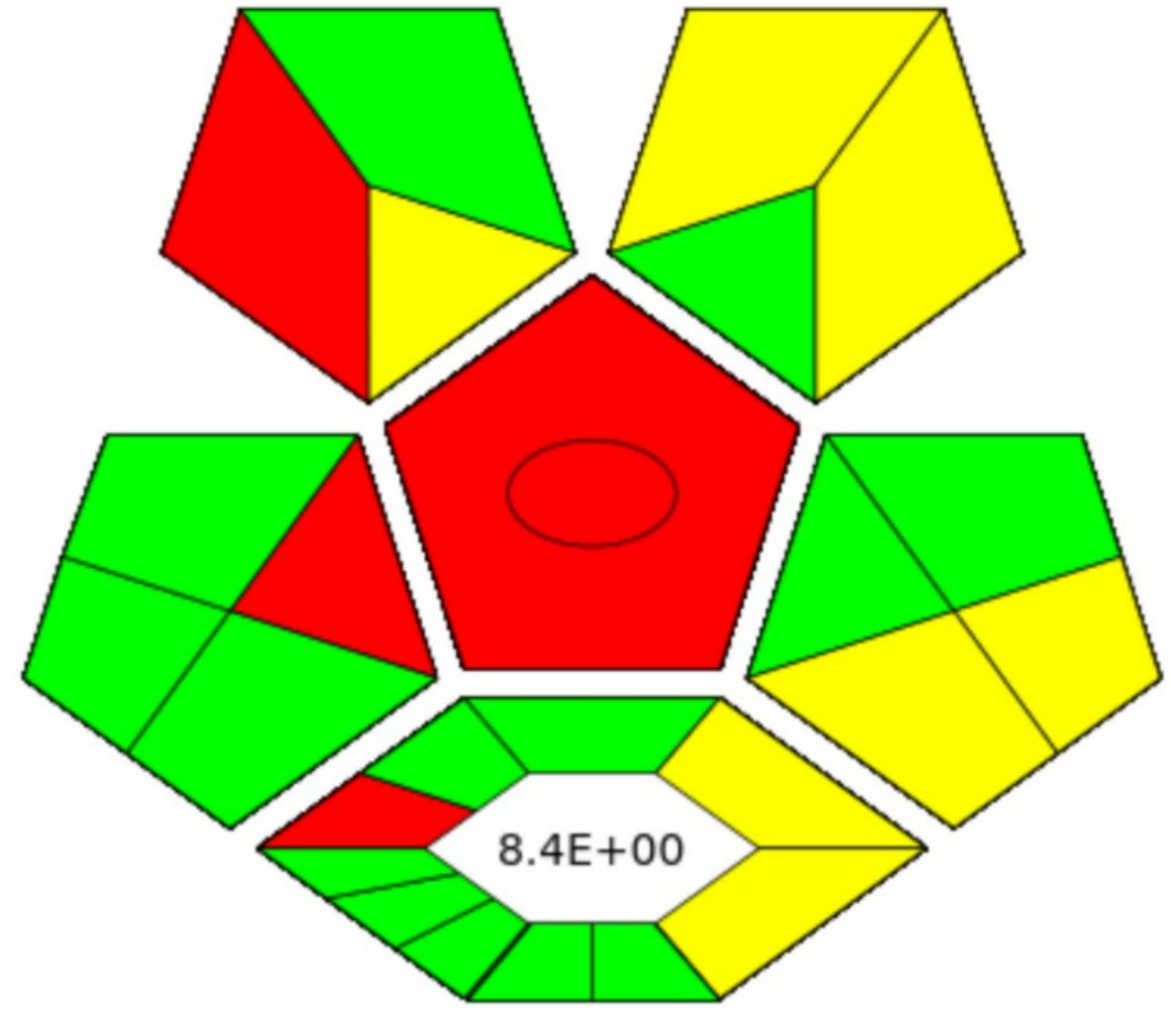
Green assessment for the proposed HPLC-FLD method utilizing ComplexMoGAPI method.

The sustainability of the analytical method was assessed using the complex modified green analytical procedure index (ComplexMoGAPI), which evaluates key factors such as reagent use, energy efficiency, waste production, and potential hazards, providing a comprehensive view of its environmental impact^30,56^. The assessment confirmed the method’s overall greenness in terms of performance and waste management. However, liquid–liquid extraction (LLE) showed limitations in its sustainability, as presented in (Fig. 4).

## Conclusion

In conclusion, the developed method for the simultaneous quantification of bisoprolol, amlodipine, telmisartan, and atorvastatin in human plasma is both efficient and reliable. Its accuracy, precision, and sensitivity were confirmed through comprehensive validation, adhering to ICH guidelines. The method’s robustness and minimal environmental impact further emphasize its practical applicability in clinical and pharmacological studies. By offering a fast and cost-effective solution, this method holds significant potential for routine therapeutic drug monitoring, ensuring better management of cardiovascular treatments while supporting sustainable analytical practices.

## Data Availability

The datasets used and/or analyzed during the current study are available from the corresponding author on reasonable request.
